# Octanoic Acid‐Rich Enteral Nutrition Regulates Intestinal M1/M2 Macrophage Polarization via the PPARγ/STAT‐1/STAT‐6 Pathway to Alleviate Inflammatory Bowel Disease

**DOI:** 10.1002/fsn3.71234

**Published:** 2025-11-17

**Authors:** Liang Xue, Chun Cao

**Affiliations:** ^1^ Department of Surgical Oncology, the First People's Hospital of Lianyungang Lianyungang China; ^2^ The Affiliated Lianyungang Hospital of Xuzhou Medical University Lianyungang China; ^3^ Department of General Surgery Second Affiliated Hospital of Soochow University Suzhou China

**Keywords:** enteral nutrition, inflammatory bowel disease, macrophage polarization, octanoic acid, PPARγ/STAT‐1/STAT‐6

## Abstract

Inflammatory bowel disease (IBD) is a complex chronic intestinal inflammatory disease involving immune, genetic and environmental interactions. A disrupted balance in the polarization of intestinal macrophages is a key pathogenic link. This study explored whether octanoic acid (OA)‐rich enteral nutrition (EN) regulated intestinal M1/M2 macrophage polarization via the PPARγ/STAT‐1/STAT‐6 pathway to alleviate IBD. First, to assess the effects of OA‐rich EN on IBD, four groups were established: the sham, IBD, IBD + EN, and IBD + OA‐rich EN. Second, to verify the regulatory role of intestinal M1/M2 macrophage polarization via the PPARγ/STAT‐1/STAT‐6 pathway, six groups were established: the sham, IBD, IBD + OA‐rich EN, IBD + OA‐rich EN with intraperitoneal injections of IFNγ, AS1517499, or SR202. RAW264.7 cells were also used to observe how OA influences LPS/IFNγ‐induced M1 polarization. OA‐rich EN notably alleviated IBD symptoms, activated the PPARγ/STAT‐1/STAT‐6 pathway and remodeled the M1/M2 polarization balance of intestinal macrophages. These effects were greater than those in the IBD + EN group. Blocking the activation of PPARγ, activating STAT‐1 or inhibiting STAT‐6 reversed the protective effect of OA‐rich EN on remodeling the M1/M2 polarization balance of intestinal macrophages and IBD symptoms. In vitro experiments further confirmed that OA regulated macrophage polarization via the PPARγ/STAT‐1/STAT‐6 pathway. This is the first confirmation that OA‐rich EN alleviates IBD by activating PPARγ/STAT‐1/STAT‐6 to regulate macrophage polarization, highlighting its potential as a nutritional therapy for IBD.

## Graphical abstract

Octanoic acid‐rich enteral nutrition could regulate the M1/M2 polarization of intestinal macrophages and simultaneously repair the intestinal mucosal barrier by activating the PPARγ/STAT‐1/STAT‐6 pathway, thereby significantly alleviating inflammatory bowel disease‐induced intestinal injury.

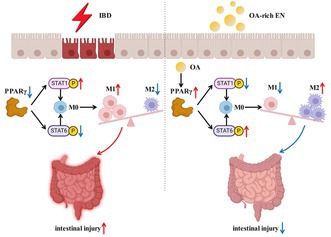

## 1 Introduction

Inflammatory bowel disease (IBD) encompasses a group of chronic, non‐specific intestinal inflammatory disorders, including Crohn's disease and ulcerative colitis. IBD not only makes patients suffer from abdominal pain, diarrhea, and hematochezia but also causes serious complications, such as intestinal obstruction, intestinal perforation, and toxic megacolon (Gordon et al. 2023). The pathogenesis of IBD is generally considered to be the result of multiple interacting factors, including genetic susceptibility, environmental triggers, intestinal microbiota imbalance, and abnormal activation of the immune system, among which gut immune system disorders, especially an uncontrolled intestinal inflammatory response, are considered the key link in the pathogenesis of IBD (Gilliland et al. 2024; Singh and Bernstein 2022; Saez et al. 2023).

As the core component of innate immunity, intestinal macrophages play critical roles in immune regulation and inflammation (Saez et al. 2023). Intestinal macrophages exhibit a high degree of plasticity and can polarize into M1 and M2 macrophages depending on the microenvironmental stimuli. When stimulated with lipopolysaccharide (LPS) and interferon‐γ (IFNγ), M1‐type macrophages are rapidly activated and secrete large quantities of pro‐inflammatory cytokines such as tumor necrosis factor‐α (TNF‐α), interleukin‐1β (IL‐1β) and IL‐6 to mediate a strong inflammatory response (Tian et al. 2021). In contrast, M2 macrophages are primarily responsible for the secretion of anti‐inflammatory cytokines and arginase‐1 (Arg‐1), which play roles in tissue repair and immune regulation (Na et al. 2019). The balance of M1 and M2 polarization is disrupted in IBD patients. Elevated levels of pro‐inflammatory factors in the intestinal microenvironment trigger overactivation of M1 macrophages, which continuously release large amounts of pro‐inflammatory cytokines and damage the intestinal tract (Zhang et al. 2023). Therefore, regulating the polarization of intestinal macrophages and restoring intestinal immunity are promising therapeutic strategies for IBD.

The signal transducer and activator of transcription (STAT) family is crucial for cell signal transduction and gene transcription regulation. Among them, STAT‐1 and STAT‐6 are important in macrophage polarization (Wu et al. 2021; Satoh et al. 2010). When IFNγ binds to its receptor, it activates receptor‐associated tyrosine kinase to phosphorylate STAT‐1, and phosphorylated STAT‐1 forms a synchronous dimer and translocates into the nucleus, where it initiates the transcription of genes related to M1‐type macrophages and promotes the polarization of macrophages toward the M1 type (Wu et al. 2021). In contrast, STAT‐6 is a key molecule in the IL‐4/IL‐13 pathway. Under stimulation by IL‐4/IL‐13, STAT‐6 is activated and then enters the nucleus to drive macrophages to polarize toward the M2 type (Satoh et al. 2010). In the pathological state of IBD, STAT‐1 is excessively activated, whereas STAT‐6 is relatively inhibited, resulting in an imbalance in M1/M2 polarization and exacerbated inflammation (Xue and Wu 2025). In our previous study, peroxisome proliferator‐activated receptor γ (PPARγ) activation reduced the expression of M1 macrophage‐related genes by inhibiting IFNγ‐induced STAT‐1 phosphorylation, thereby suppressing M1 polarization. Moreover, PPARγ activation can increase IL‐4/IL‐13‐induced STAT‐6 phosphorylation and promote the polarization of macrophages toward the M2 type (Xue and Wu 2025). Therefore, activating PPARγ has become a potential way to regulate M1/M2 polarization and alleviate IBD via the STAT‐1/STAT‐6 pathway.

Nutritional intervention is vital in IBD treatment. Enteral nutrition (EN) not only provides patients with the energy needed to maintain life activities but also preserves intestinal mucosal integrity and regulates intestinal immune function (Bischoff et al. 2020). As a medium‐chain fatty acid, octanoic acid (OA), which is commonly present in dairy products, coconut oil, and nuts in nature, has gained widespread attention in the study of critical illness (Tang et al. 2025, 2023a, 2023b; Zhang et al. 2024). OA exhibits various biological effects, including anti‐inflammatory, anti‐oxidant, and the ability to regulate immunity and intestinal flora (Tang et al. 2025). OA‐rich EN has been developed on the basis of the unique biological characteristics of OA. Previous studies on sepsis have shown that OA‐rich EN can be rapidly absorbed by the intestinal tract and activate PPARγ in the intestine and liver, thereby alleviating acute intestinal and liver injury (Tang et al. 2023a, 2023b). Given that sepsis and IBD share common pathological features including excessive inflammatory responses and intestinal barrier damage, the protective effect of OA‐rich EN in sepsis suggests its potential to mitigate similar pathological processes in IBD by targeting overlapping molecular pathways such as PPARγ‐mediated immune regulation. In addition, OA can also maintain the energy supply of intestinal mucosal epithelial cells, enhance tight junction protein expression, facilitate repair of the impaired intestinal mucosal barrier, decrease intestinal permeability, and mitigate antigen exposure, thereby indirectly inhibiting the excessive activation of intestinal macrophages (Wang et al. 2018).

This study focused on the therapeutic effect of the nutritional nutrient OA on IBD from the perspective of the activation of PPARγ/STAT‐1/STAT‐6 in the regulation of intestinal M1/M2 macrophage polarization, aiming to provide a solid theoretical basis for novel IBD treatment methods based on nutritional intervention.

## 2 Materials and Methods

### 2.1 Animal Experiments

Eighty adult male C57BL/6 mice (weighing 19.5–21.6 g) were procured from Soochow University (Suzhou, China). The mice were kept under standard environmental conditions, with free access to food and drinking water. The Research Ethics Committee of Soochow University granted approval for all experimental procedures.

First, to explore the effect of OA‐rich EN on IBD, four groups (*n* = 8 per group) were established: the sham, IBD, IBD + EN, and IBD + OA‐rich EN groups. The gastric tube was inserted for EN administration before the study and the detail was described in the previous study (Tang et al. 2023a, 2023b; Zhang et al. 2024). To establish an experimental murine model of IBD, the mice were provided drinking water containing 2.5% dextran sulfate sodium salt (DSS, MP Biomedicals, USA) for 7 consecutive days, followed by regular drinking water for 2 days. The mice in the IBD + EN and IBD + OA‐rich EN groups received EN (10 kcal/day, Ensure Plus, Abbott Laboratories, Chicago, USA) or OA‐rich EN (9.55 kcal/day of EN and 0.45 kcal/d of OA, Aladdin, chemical abstracts service registry number: 124–07‐2, purity ≥ 99%, China) via a gastric tube for 9 days. The total caloric intake of the mice in these groups was comparable to that of the IBD group. Second, to verify the regulatory role of intestinal M1/M2 macrophage polarization via the PPARγ/STAT‐1/STAT‐6 pathway, six groups (*n* = 8 per group) were established: the sham, IBD, IBD + OA‐rich EN, IBD + OA‐rich EN + interferonγ (IFNγ), IBD + OA‐rich EN + AS1517499 (AS, a STAT‐6 inhibitor), and IBD + OA‐rich EN + SR202 (a PPARγ inhibitor). All mice in the OA‐rich EN‐containing groups received the same OA‐rich EN. The mice in the OA‐rich EN + IFNγ, IBD + OA‐rich EN + AS, and IBD + OA‐rich EN + SR202 groups were intraperitoneally injected with IFNγ (20 μg/mouse, MedChemExpress, China), AS (10 mg/kg/every other day, MedChemExpress, China), or SR202 (3 mg/kg/day, APExBIO, USA), respectively. The mice were individually housed in separate cages, with daily monitoring of body weight fluctuations, stool consistency, and hematochezia. From Days 0 to 9, the disease activity index (DAI) was evaluated on the basis of these parameters. On Day 10, pentobarbital sodium was used for anesthesia, followed by collection of the entire colon and serum.

### 2.2 Cell Culture and Treatment

Abbkine Scientific Co. Ltd. supplied the RAW264.7 cells used in this study. Cultivation was performed in Dulbecco's modified Eagle's medium, with the addition of 10% fetal bovine serum and 1% penicillin–streptomycin solution under standard culture conditions. To induce M1 polarization of macrophages, lipopolysaccharide (LPS, 15 ng/mL) and IFNγ (50 ng/mL) were added to the culture medium. OA (100 μM) and/or SR202 (0.4 mM) were added to the culture medium. The doses of OA and SR202 were based on the previous study (Tang et al. 2023a, 2023b). After 24 h of incubation with LPS/IFNγ, OA, or SR202, RAW264.7 cells were harvested to measure protein and mRNA expression.

### 2.3 Hematoxylin–Eosin Staining and Scores

Colon tissue samples were collected and immediately immersed in 4% paraformaldehyde for fixation, followed by paraffin embedding. A Leica RM2235 rotary microtome (Leica Microsystems, Wetzlar, Germany) was used to prepare 5‐μm‐thick sections. Then sections were stained with Hematoxylin–Eosin (H&E). Morphological alterations in mucosal architecture, including the density of inflammatory cell infiltration, depth of tissue injury, degree of crypt damage, and percentage of affected area, were quantitatively assessed via ordinary light microscopy.

### 2.4 Periodic Acid Schiff Staining

Sections were dewaxed in xylene and rehydrated, followed by oxidation with periodic acid solution for 10 min. Next, they were incubated with Schiff reagent for 15 min in the dark, followed by thorough triple‐distilled water washing. For nuclear counterstaining, sections were stained with Mayer's hematoxylin, rinsed, and differentiated with acidified ethanol. After being blued in ammonia water, sections were dehydrated and cleared in xylene. Goblet cell mucin distribution was assessed via ordinary light microscopy.

### 2.5 Immunofluorescence Analysis

Colon tissue sections were heated in citrate buffer (10 mM, pH 6.0) at 95°C–98°C for 20 min to facilitate optimal antigen exposure. Sections were incubated overnight at 4°C with primary antibodies against F4/80 (Cell Signaling Technology, CST, 1:200), Arg‐1 (CST, 1:100), and iNOS (Abcam, 1:100). After three washes with phosphate‐buffered saline to remove unbound primary antibodies, sections were incubated with Cy3/FITC‐conjugated secondary antibodies (1:200) for 1 h in the dark. Nuclei were counterstained with 4′,6‐diamidino‐2‐phenylindole (1:1000) for 5 min to visualize the cell nuclei. Images were acquired via fluorescence microscopy (Carl Zeiss MicroImaging GHBH, Germany).

### 2.6 Immunohistochemical Staining

After antigen retrieval, endogenous peroxidase activity was blocked with 3% H_2_O_2_, followed by protein blocking with 10% normal goat serum. Overnight incubation (16 h at 4°C) with primary antibodies against ZO‐1 (Proteintech, 1:100) and occludin (CST, 1:100) was conducted and then sequentially treated with a biotinylated secondary antibody (1:200) and HRP‐streptavidin. DAB chromogen was used to develop the signal, Mayer's hematoxylin was used for nuclear counterstaining, and visualization was performed via light microscopy.

### 2.7 Western Blotting Analysis

Protein lysates of colon tissue and RAW264.7 cells were prepared via ice‐cold radioimmunoprecipitation lysis buffer supplemented with protease and phosphatase inhibitors. Proteins were separated by SDS–PAGE on bis‐acrylamide resolving gels using Tris–glycine–SDS running buffer at 120 V. Proteins were transferred to PVDF membranes via semidry transfer, and then membranes were blocked. Primary antibodies were against ZO‐1 (Proteintech, 1:1000), occludin (CST, 1:1000), iNOS (Abcam, 1:1000), Arg‐1 (CST, 1:1000), PPARγ (Abcam, 1:1000), STAT‐1/p‐STAT‐1 (CST, 1:1000), STAT‐6/p‐STAT‐6 (CST, 1:1000), and β‐actin (CST, 1:5000). Membranes were then probed with the corresponding secondary antibody. An enhanced chemiluminescence substrate was used to detect membranes on a Bio‐Rad ChemiDoc MP system, while densitometric analysis was performed with Image Lab software.

### 2.8 Real‐Time Polymerase Chain Reaction

Total RNA was extracted from colon tissue and RAW264.7 cells. Quantitative PCR was performed with PowerUp SYBR Green Master Mix on a StepOnePlus system using gene‐specific primers (Table 1). Reactions were run in technical triplicates under the following conditions: 50°C for 2 min, 95°C for 2 min, and then 40 cycles of 95°C for 15 s and 60°C for 1 min. Target gene expression was calculated and normalized to that of β‐Actin.

**TABLE 1:** The primer of the target gene.

Target gene		Primer
Arg‐1	Forward	5′‐CTCCAAGCCAAAGTCCTTAGAG‐3′
	Reverse	5′‐GGAGCTGTCATTAGGGACATCA‐3′
Fizz 1	Forward	5′‐CCAATCCAGCTAACTATCCCTCC‐3′
	Reverse	5′‐ACCCAGTAGCAGTCATCCCA‐3′
Ym 1	Forward	5′‐CAGGTCTGGCAATTCTTCTGAA‐3′
	Reverse	5′‐GTCTTGCTCATGTGTGTAAGTGA‐3′
iNOS	Forward	5′‐GGAGTGACGGCAAACATGACT‐3′
	Reverse	5′‐TCGATGCACAACTGGGTGAAC‐3′
β‐Actin	Forward	5′‐ACAGAGCCTCGCCTTTGCCGAT‐3′
	Reverse	5′‐GACCCATGCCCACCATCACGC‐3′

### 2.9 Statistical Analysis

All statistical analyses were performed via GraphPad Prism software (version 10.42; GraphPad Software Inc., San Diego, CA, USA). Data are expressed as the mean ± standard error of the mean. Multiple group comparisons were analyzed using one‐way analysis of variance followed by Tukey's honestly significant difference post hoc test. Statistical significance was set at *p* < 0.05, and all tests were two‐tailed.

## 3 Results

### 3.1 OA‐Rich EN Significantly Alleviated Clinical Symptoms and Intestinal Injury in IBD Mice

**FIGURE 1:**
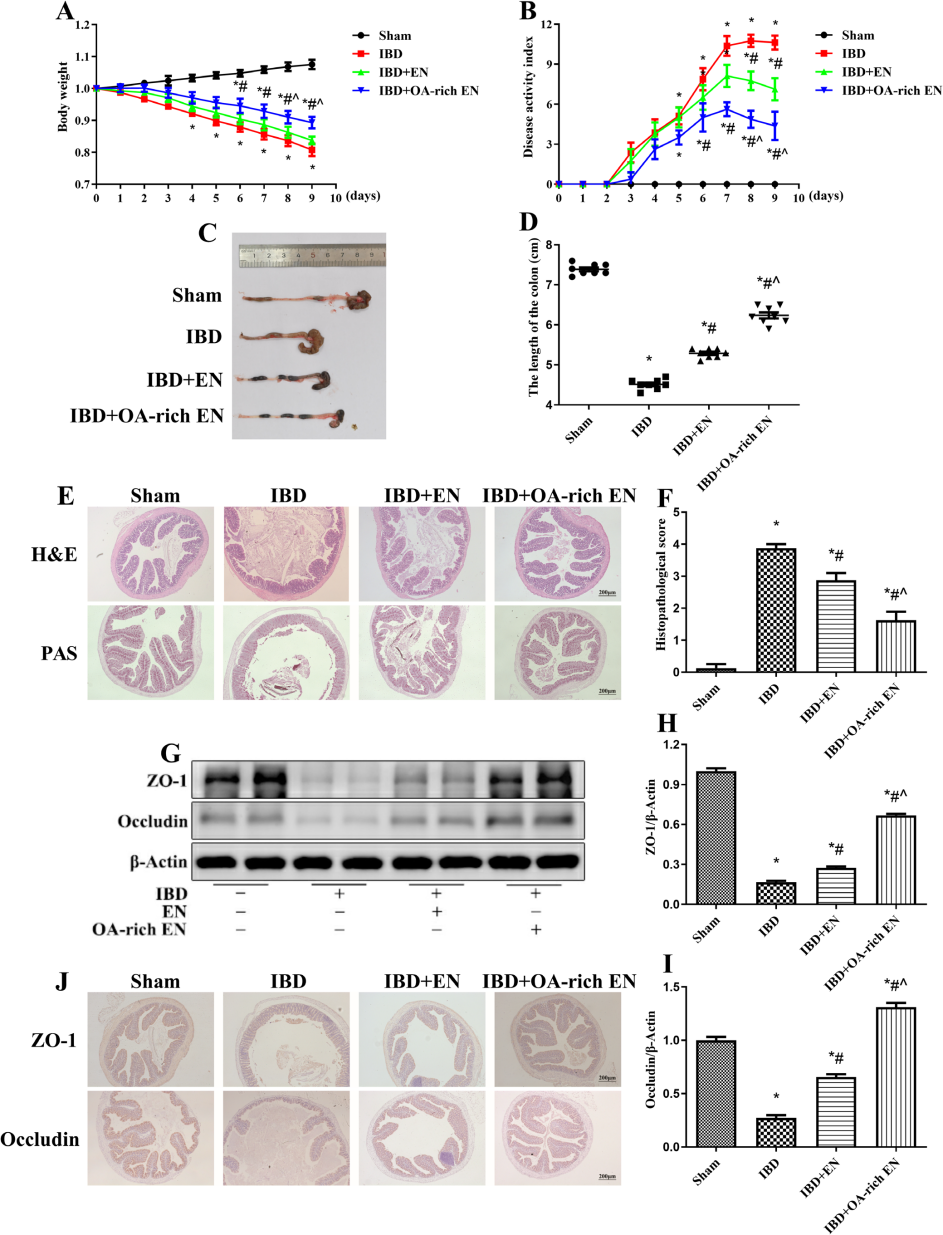
OA‐rich EN significantly alleviated clinical symptoms and intestinal injury in IBD mice. The weights of the mice were assessed daily (A). The evaluation of the disease activity index score (B). Images of a representative colon (C) and the length of the colon were measured (D). Pathomorphology in the colon was evaluated via hematoxylin–eosin (H&E) staining (E) and the score of the colon (F). The goblet cell density, glycoprotein content and mucus secretion in the colon were evaluated via Periodic acid Schiff (PAS) staining (G). The expression of ZO‐1 and occludin was measured via western blotting (G–I) and immunohistochemistry (J). The data are presented as the mean ± standard error of the mean. *p < 0.05 vs. the sham group; #*p* < 0.05 vs. the IBD group; ^*p* < 0.05 vs. the IBD + EN group.

IBD group presented significant body weight loss, bloody stools, and diarrhea, and the DAI score significantly increased after modeling. After OA‐rich EN intervention, the body weight loss of the mice was reduced, the symptoms of bloody stool and diarrhea were significantly relieved, and the DAI score was significantly lower than that of the IBD group, whereas the ability of EN alone to improve the disease activity was weaker than that of the OA‐rich EN group (Figure 1A–D).

Pathomorphology revealed that colon contracture, a disordered arrangement of colon epithelial cells and intestinal glands in the mucosal layer, and a large number of neutrophils and lymphocytes infiltrating the colon mucosa and submucosa were observed in the IBD group. After OA‐rich EN intervention, inflammatory cell infiltration and crypt damage were decreased, and the effects were greater than those in the IBD + EN group (Figure 1E,F). The IBD group showed significantly lower goblet cell count, glycoprotein content, and mucus secretion compared with the sham group; OA‐rich EN restored the number of goblet cells, glycoprotein content and mucus secretion to levels equivalent to those in the sham group (Figure 1E).

The expression of tight junction proteins in the IBD group was markedly reduced. After OA‐rich EN intervention, the expression of ZO‐1 and occludin was significantly increased, which was significantly greater than EN alone (Figure 1G–I). Moreover, immunohistochemistry further confirmed that the localization of ZO‐1 and occludin on the cell membrane was more continuous in the OA‐rich EN group than in the IBD group, suggesting that OA‐rich EN was capable of maintaining intestinal barrier integrity (Figure 1J). These results indicate that the addition of OA can enhance the therapeutic effect of EN on IBD.

### 3.2 OA‐Rich EN Activated the PPARγ/STAT‐1/STAT‐6 Pathway and Regulated the Balance of M1/M2 Macrophage Polarization

**FIGURE 2:**
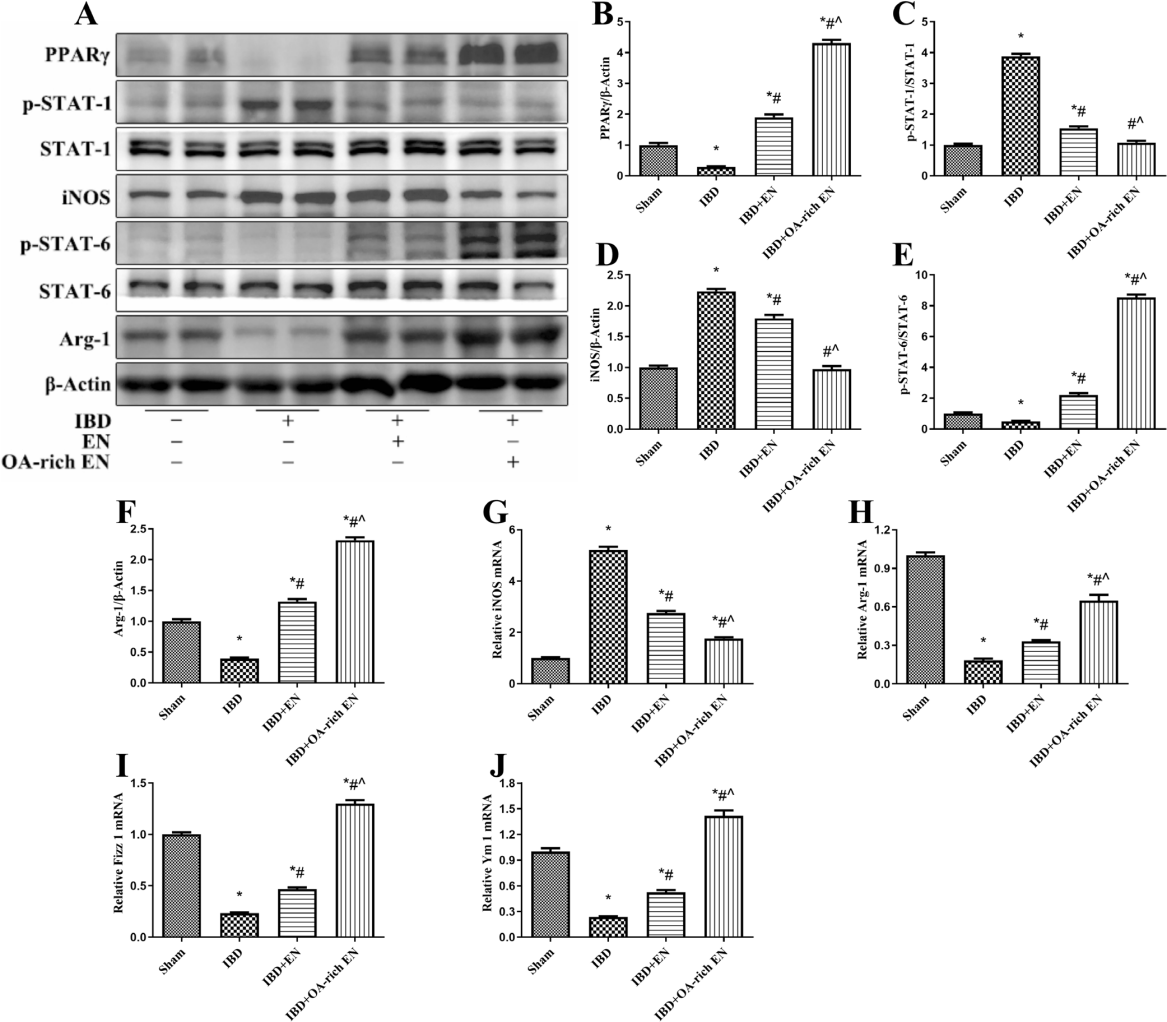
OA‐rich EN activated the PPARγ/STAT‐1/STAT‐6 pathway and regulated the balance of M1/M2 macrophage polarization in IBD mice. The protein expression of PPARγ, p‐STAT‐1, STAT‐1, iNOS, PPARγ, p‐STAT‐6, STAT‐6, and Arg‐1 was measured via western blotting (A). The relative protein expression of PPARγ (B), iNOS (D), and Arg‐1 (F) was normalized to that of β‐Actin. The protein expression of p‐STAT‐1 was normalized to that of STAT‐1 (C). The protein expression of p‐STAT‐6 was normalized to that of STAT‐6 (E). The mRNA expression of iNOS (G), Arg‐1 (H), Fizz 1 (I), and Ym 1 (J) was measured via real‐time polymerase chain reaction. The expression of each mRNA was normalized to that of β‐Actin. The data are presented as the mean ± SE of the mean. *p < 0.05 vs. the sham group; #*p* < 0.05 vs. the IBD group; ^*p* < 0.05 vs. the IBD + EN group.

**FIGURE 3:**
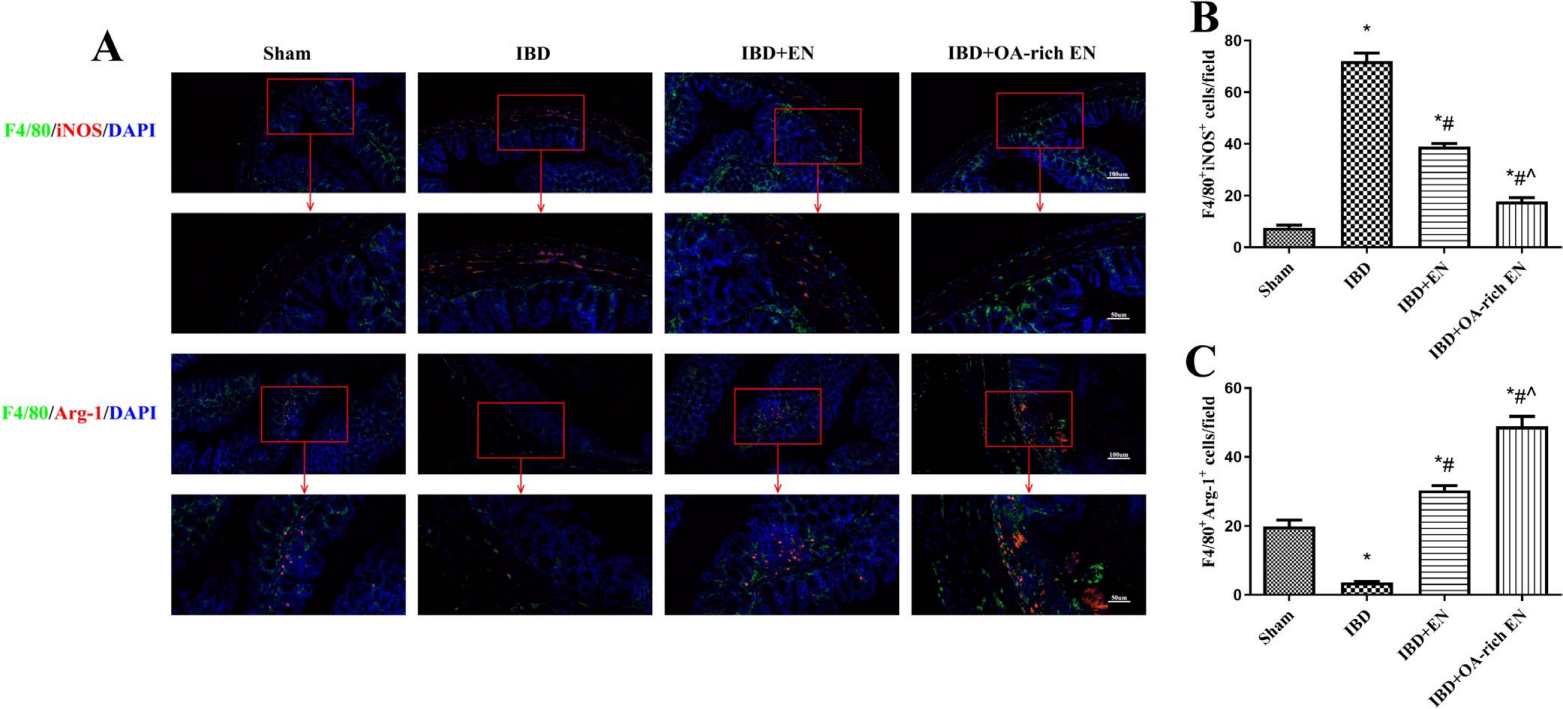
OA‐rich EN modulated the plasticity of intestinal macrophages in IBD mice. Immunofluorescence images of F4/80, iNOS, Arg‐1, and DAPI in the colon (A). The number of F4/80^+^ iNOS^+^ cells was quantified (B). The number of F4/80^+^ Arg‐1^+^ cells were quantified (C). The data are presented as the mean ± standard error of the mean. *p < 0.05 vs. the sham group; #*p* < 0.05 vs. the IBD group > *p*; ^*p* < 0.05 vs. the IBD + EN group.

DSS treatment significantly inhibited PPARγ, promoted STAT‐1 phosphorylation, inhibited STAT‐6 phosphorylation, increased iNOS expression, and decreased the expression of Arg‐1, Fizz 1, and Ym 1, suggesting that IBD promoted the polarization of macrophages toward the M1 type via the PPARγ/STAT‐1/STAT‐6 pathway. OA‐rich EN significantly activated PPARγ, inhibited STAT‐1 phosphorylation, promoted STAT‐6 phosphorylation, reduced iNOS expression, and up‐regulated the expression of Arg‐1, Fizz 1, and Ym 1, and these effects were greater than those in the IBD + EN group (Figure 2). Immunofluorescence also revealed that significantly more M1‐type macrophage marker F4/80^+^ iNOS^+^ positive cells and significantly fewer M2‐type marker F4/80^+^ Arg‐1^+^ positive cells were in the IBD group than in the sham group. OA‐rich EN intervention led to a reduction in F4/80^+^ iNOS^+^ cells and an increase in F4/80^+^ Arg‐1^+^ cells, indicating that OA‐rich EN can activate the PPARγ/STAT‐1/STAT‐6 pathway and drive macrophages to polarize toward the M2 type (Figure 3).

### 3.3 The Protective Effect of OA‐Rich EN on IBD Was Dependent on M1/M2 Macrophage Polarization via the PPARγ/STAT‐1/STAT‐6 Pathway

**FIGURE 4:**
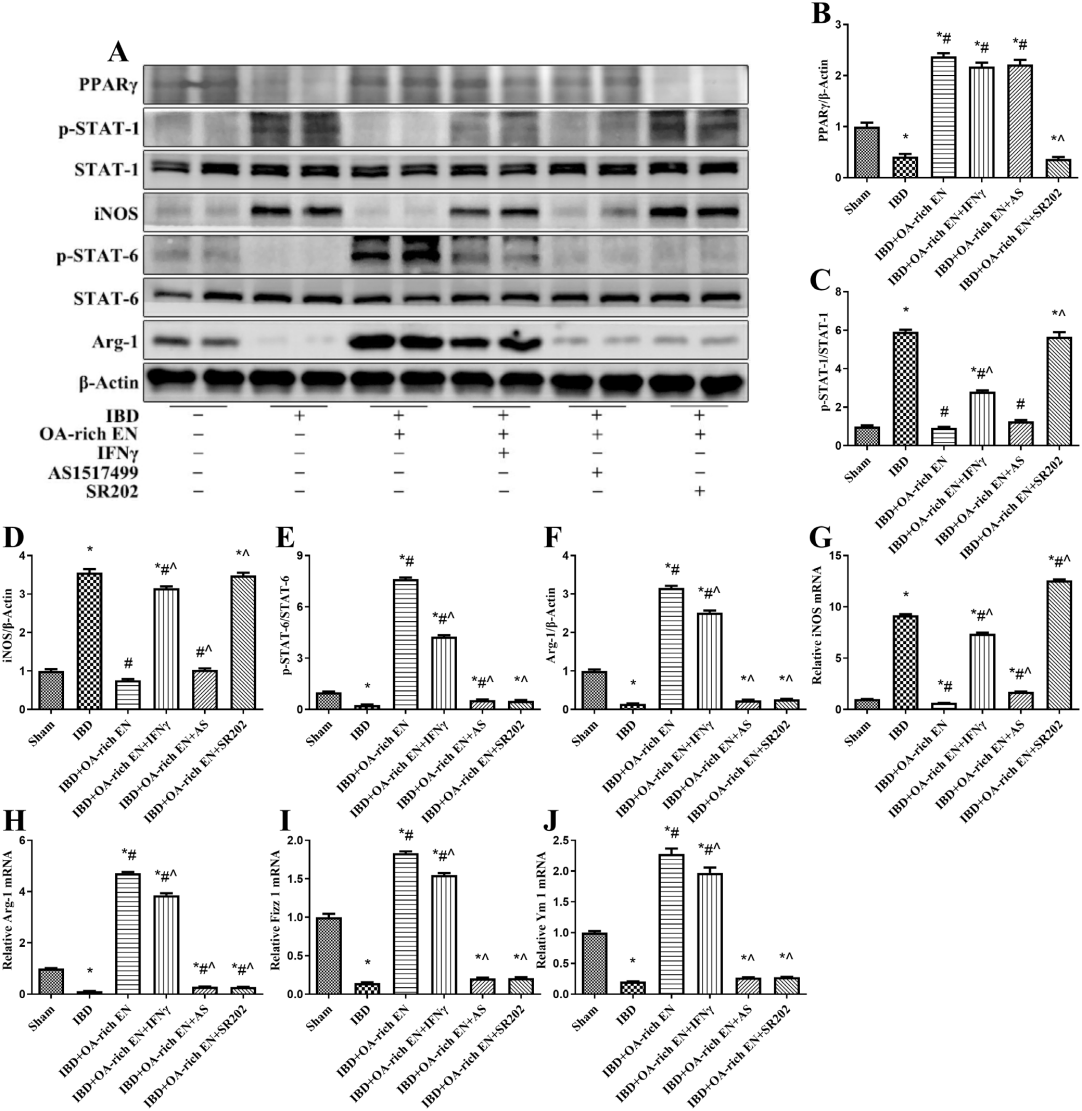
OA‐rich EN regulated M1/M2 macrophage polarization via the PPARγ/STAT‐1/STAT‐6 pathway in IBD mice. The protein expression of PPARγ, p‐STAT‐1, STAT‐1, iNOS, PPARγ, p‐STAT‐6, STAT‐6, and Arg‐1 was measured via western blotting (A). The relative protein expression of PPARγ (B), iNOS (D), and Arg‐1 (F) was normalized to that of β‐actin. The protein expression of p‐STAT‐1 was normalized to that of STAT‐1 (C). The protein expression of p‐STAT‐6 was normalized to that of STAT‐6 (E). The mRNA expression of iNOS (G), Arg‐1 (H), Fizz 1 (I), and Ym 1 (J) was measured via real‐time polymerase chain reaction. The expression of each mRNA was normalized to that of β‐Actin. The data are presented as the mean ± standard error of the mean. *p < 0.05 vs. the sham group; #*p* < 0.05 vs. the IBD group; ^*p* < 0.05 vs. the IBD + OA‐rich EN group.

**FIGURE 5:**
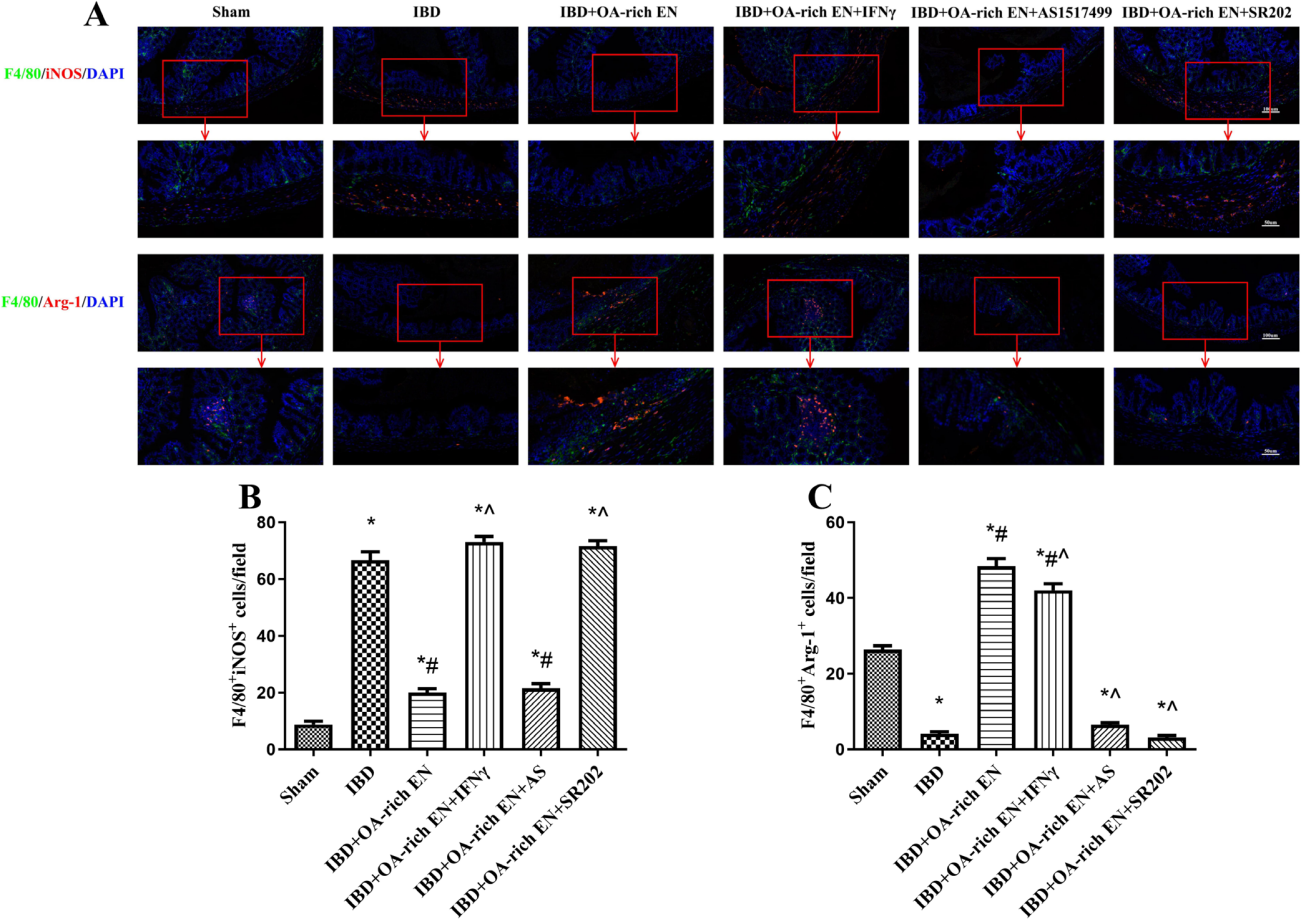
Immunofluorescence validation of the effects of pathway inhibitors on macrophage polarization. Immunofluorescence images of F4/80, iNOS, Arg‐1, and DAPI in the colon (A). The number of F4/80^+^ iNOS^+^ cells was quantified (B). The number of F4/80^+^ Arg‐1^+^ cells were quantified (C). The data are presented as the mean ± standard error of the mean. *< 0.05 vs. the sham group>*p*; #*p* < 0.05 vs. the IBD group; ^*p* < 0.05 vs. the IBD + OA‐rich EN group.

The application of IFNγ (a STAT‐1 activator) or AS1517499 (a STAT‐6 inhibitor) reversed the inhibitory effect of OA‐rich EN on M1 polarization and its promotional effect on M2 polarization. Moreover, the PPARγ inhibitor SR202 reversed the regulatory effect of OA‐rich EN on the STAT‐1/STAT‐6 pathway and M1/M2 macrophage polarization (Figures 4 and 5).

**FIGURE 6:**
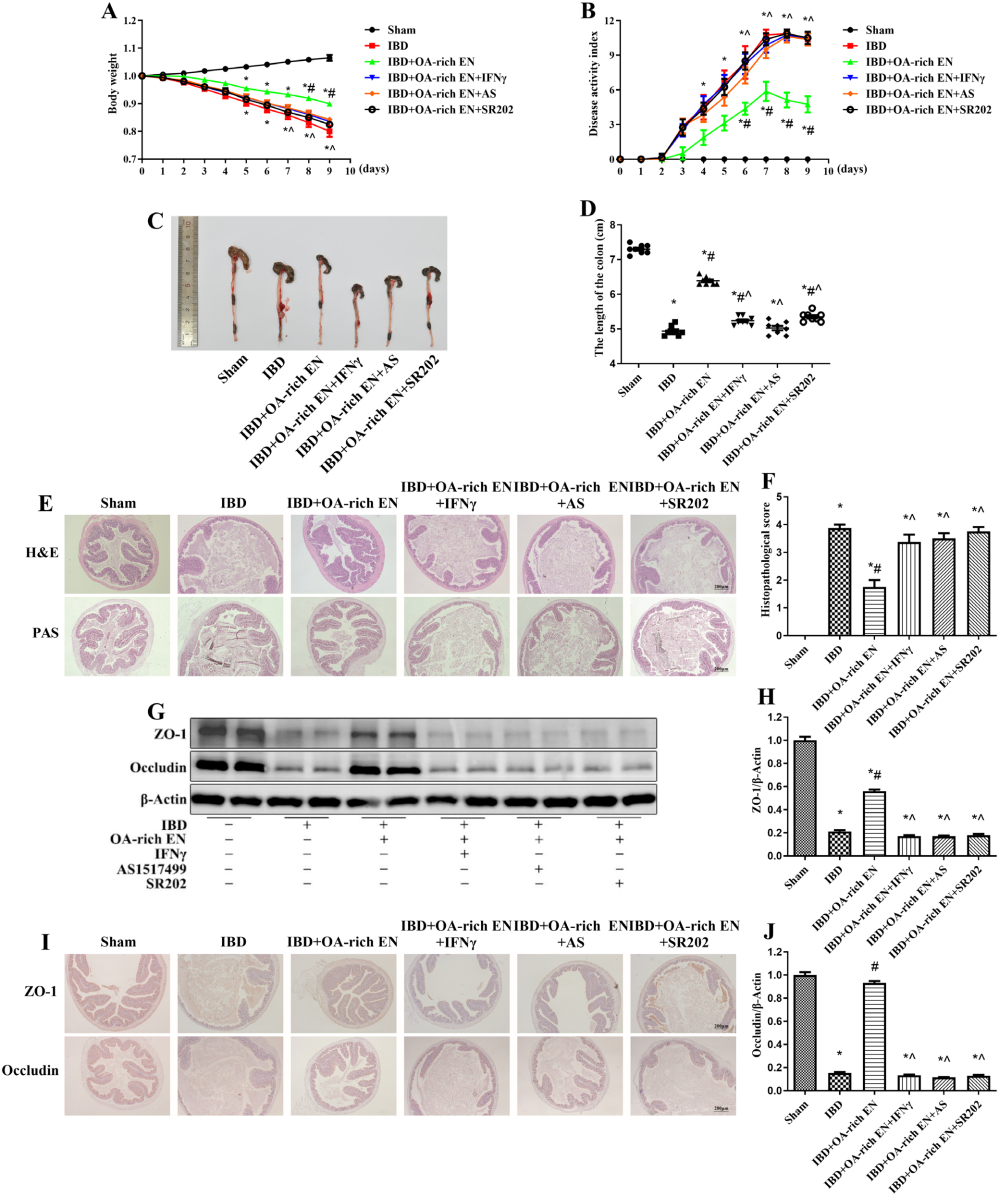
The protective effect of OA‐rich EN on IBD was dependent on M1/M2 macrophage polarization via the PPARγ/STAT‐1/STAT‐6 pathway. The weights of the mice were assessed daily (A). The evaluation of the disease activity index score (B). Images of a representative colon (C) and the length of the colon were measured (D). Pathomorphology in the colon was evaluated via hematoxylin–eosin (H&E) staining (E) and the score of the colon (F). The goblet cell density, glycoprotein content, and mucus secretion in the colon were evaluated via periodic acid Schiff (PAS) staining (G). The expression of ZO‐1 and occludin was measured via western blotting (G–I) and immunohistochemistry (J). The data are presented as the mean ± standard error of the mean. *p < 0.05 vs. the sham group; #*p* < 0.05 vs. the IBD group; ^*p* < 0.05 vs. the IBD + OA‐rich EN group.

The body weight loss, DAI score, and degree of intestinal injury in the IBD + OA‐rich EN + IFNγ, IBD + OA‐rich EN + AS and IBD + OA‐rich EN + SR202 groups were significantly greater than those in the IBD + OA‐rich EN group (Figure 6A–F), and the expression of tight junction proteins decreased again (Figure 6G–I). These findings suggest that the protective effect of OA‐rich EN on IBD is dependent on M1/M2 macrophage polarization via the PPARγ/STAT‐1/STAT‐6 pathway.

### 3.4 OA Regulated M1/M2 Macrophage Polarization via the PPARγ/STAT‐1/STAT‐6 Pathway In Vitro

**FIGURE 7:**
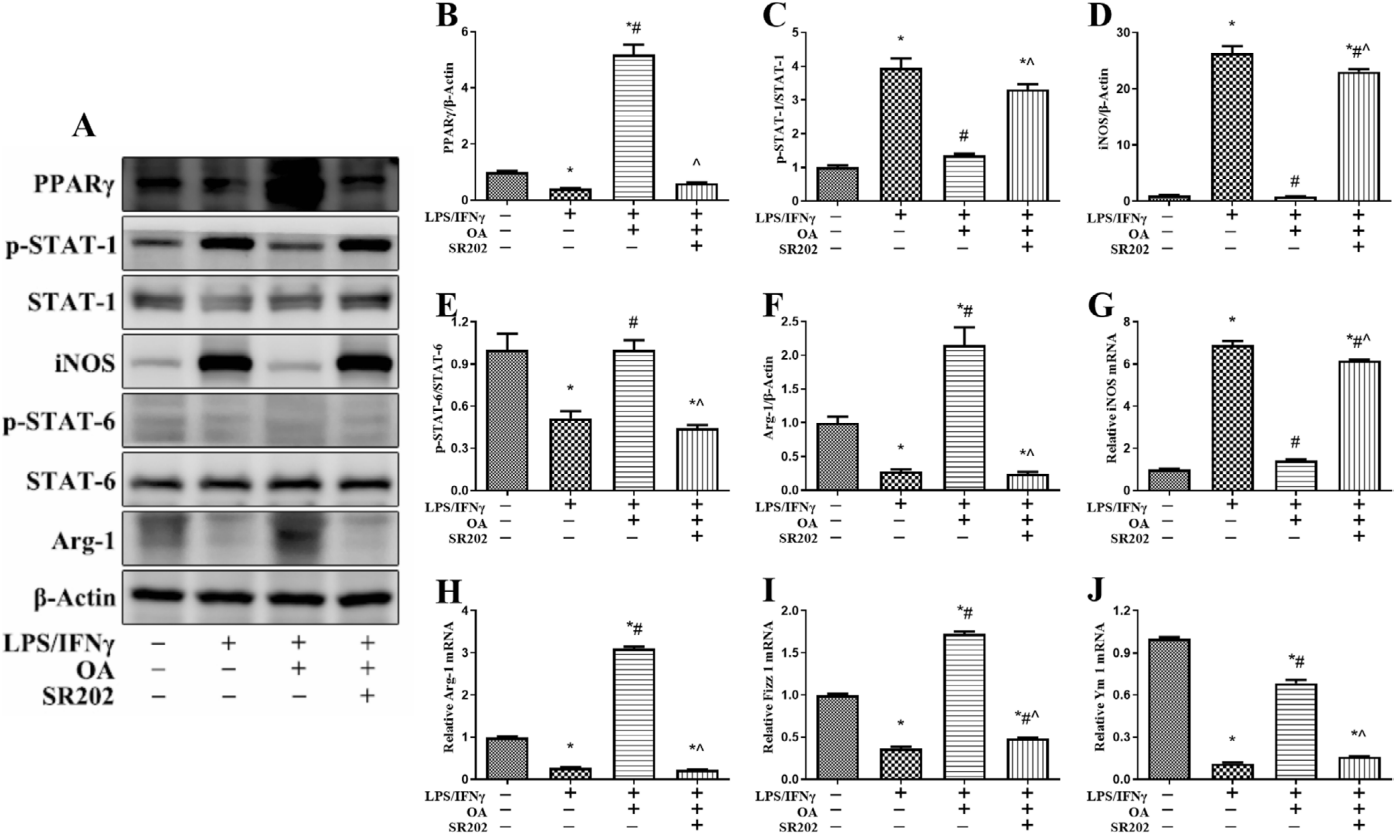
OA regulated M1/M2 macrophage polarization via the PPARγ/STAT‐1/STAT‐6 pathway in vitro. The protein expression of PPARγ, p‐STAT‐1, STAT‐1, iNOS, PPARγ, p‐STAT‐6, STAT‐6, and Arg‐1 was measured via western blotting (A). The relative protein expression of PPARγ (B), iNOS (D), and Arg‐1 (F) was normalized to that of β‐Actin. The protein expression of p‐STAT‐1 was normalized to that of STAT‐1 (C). The protein expression of p‐STAT‐6 was normalized to that of STAT‐6 (E). The mRNA expression of iNOS (G), Arg‐1 (H), Fizz 1 (I), and Ym 1 (J) was measured via real‐time polymerase chain reaction. The expression of each mRNA was normalized to that of β‐actin. The data are presented as the mean ± standard error of the mean. *p < 0.05 vs. the sham group; #*p* < 0.05 vs. the IBD group; ^*p* < 0.05 vs. the IBD + OA‐rich EN group.

After inducing M1 polarization in RAW264.7 cells with LPS/IFNγ, OA significantly activated PPARγ, inhibited STAT‐1 phosphorylation, promoted STAT‐6 phosphorylation, reduced iNOS expression, and up‐regulated Arg‐1, Fizz 1, and Ym 1 expression. SR202 could block the activation of PPARγ by OA and thus reverse its regulatory effects on the STAT‐1/STAT‐6 pathway and the M1/M2 polarization of macrophages (Figure 7).

## 4 Discussion

IBD is a complex chronic intestinal inflammatory disorder, and its pathogenesis involves interactions among immune, genetic and environmental factors. This study concentrated on the function of OA‐rich EN in regulating the polarization of intestinal macrophages. The DSS‐induced IBD model and cell experiments confirmed that OA‐rich EN remodeled the M1/M2 polarization balance of intestinal macrophages via the PPARγ/STAT‐1/STAT‐6 pathway, thereby significantly alleviating intestinal injury in IBD. This finding not only expands the understanding of nutrition–immune interactions in the pathogenesis of IBD but also provides a new perspective for the development of novel nutritional therapy strategies for IBD.

The intestinal epithelial barrier serves as the first line of defense against external pathogens and harmful substances, and its integrity is critical for maintaining intestinal microenvironment stability. Intestinal mucosal barrier dysfunction is one of the core links in the pathogenesis of IBD (Neurath 2014). DSS‐induced IBD caused reduced the expression and structural destruction of tight junction proteins between epithelial cells, leading to increased intestinal permeability. Bacteria, endotoxins and antigenic substances that were originally blocked in the intestinal lumen can pass through the damaged barrier and enter the intestinal mucosa lamina propria, thereby activating the immune system and causing excessive inflammation. In addition, colon pathology revealed that the colonic epithelial cells and intestinal glands were disordered in the mucosal layer, a large number of neutrophils and lymphocytes infiltrated mucosa and submucosa, the structure of crypts was destroyed, and ulcers formed. PAS staining further showed reduced numbers of goblet cells, decreased glycoprotein content, and impaired mucus secretion were in the IBD group. EN's protective impact on intestinal barrier function stands as the core mechanism in IBD treatment. Nutrients in the EN, such as glutamine, are important energy substances for intestinal mucosal cells and can intestinal mucosal repair and enhance mechanical barrier function (Severo et al. 2020). Dietary fiber can undergo fermentation by the intestinal flora to generate short‐chain fatty acids, which provide energy for the colonic mucosa and strengthen the biological barrier (Loy et al. 2024). In addition, by regulating intestinal immune cell function, reducing pro‐inflammatory factor release, and boosting anti‐inflammatory factor expression, EN can reduce intestinal inflammation (Reznikov and Suskind 2023). However, during the acute stage of IBD, EN preparations alone are not very effective at improving intestinal symptoms (Bischoff et al. 2020). OA reinforces the intestinal epithelial cell immune barrier by decreasing canonical histone deacetylase pathway activity, increasing intestinal β‐defensins, promoting jejunal claudin‐1 and zonula occludens protein 1 expression, and boosting mucin‐2 and mucin‐3 secretion to block bacterial translocation (Wang et al. 2018; Zhao et al. 2021; Li et al. 2023). In addition, OA‐rich EN can increase goblet cells density by activating the PPARγ/STAT‐1/MyD88 pathway, thereby improving intestinal epithelial barrier damage in septic rats (Tang et al. 2023a, 2023b). Composed of 5‐aminosalicylic acid, eicosapentaenoic acid, and OA, CLX‐103 exhibits stability in gastric juice while undergoing enzymatic hydrolysis in the intestinal tract to release active components, which allows for the improvement of ulcerative colitis with a low dose (Kandula et al. 2016). This study revealed that OA‐rich EN could significantly alleviate intestinal injury in IBD mice and the effect was superior to that of EN alone. Specifically, OA‐rich EN further alleviated weight loss than EN alone, and the symptoms of bloody stool and diarrhea were significantly relieved on the 6th day of intervention. OA‐rich EN can reduce neutrophil and lymphocyte infiltration in the colonic mucosal layer, reduce the degree of structural destruction of the crypts, and promote mucosal repair in the ulcerated area. Moreover, OA‐rich EN significantly improved DSS‐induced intestinal barrier damage by increasing intestinal tight junction proteins.

In addition to serving as an energy substrate to repair the intestinal mucosal barrier and directly enhance the structural integrity of the barrier (Andoh et al. 2000), OA can restore intestinal function by remodeling the M1/M2 polarization balance of intestinal macrophages. Intestinal immunity, especially an imbalance in macrophage polarization, is a key factor in uncontrolled inflammation in IBD (Saez et al. 2023). In IBD patients, intestinal M1‐type macrophages are excessively activated and continuously release large amounts of proinflammatory cytokines, which in turn damage the intestinal mucosal barrier, forming a vicious cycle and aggravating disease progression. In addition, the number of intestinal M2‐type macrophages in IBD patients is significantly reduced (Zhang et al. 2023). Astragaloside IV alleviates DSS‐induced colitis by downregulating STAT‐1 to inhibit macrophage M1 polarization (Tian et al. 2021). Wu‐Mei‐Wan promotes macrophage M2 polarization by activating the STAT‐6 pathway, thereby alleviating the symptoms of ulcerative colitis (Yan et al. 2022). In intestinal immunity studies, OA is capable of improving the phagocytosis of dendritic cells and up‐regulating major histocompatibility complex class II expression, thereby maintaining the antigen‐presenting function of mature dendritic cells (Tsuzuki et al. 2006). Compared with EN alone, OA‐rich EN intervention led to a reduction in F4/80^+^ iNOS^+^ cells and an increase in F4/80^+^ Arg‐1^+^ cells, indicating that OA‐rich EN can simultaneously inhibit M1 polarization and promote M2 polarization. Our previous study revealed that DSS can cause severe colon damage accompanied by decreased expression of PPARγ; activation of PPARγ via pioglitazone can effectively facilitate macrophages to switch from M1 to M2 phenotype via the STAT‐1/STAT‐6 pathway, which can alleviate the symptoms of IBD (Xue and Wu 2025). Macrophage‐specific PPARγ deficiency significantly increases the expression of inflammatory and metabolic genes and exacerbates the symptoms of IBD (Hontecillas et al. 2011). PPARγ activation via 15d‐PGJ2 can promote the polarization of macrophages from M1 to M2 phenotype and inhibit cytokine release and neutrophil migration (Abdalla et al. 2020). OA‐rich EN can be rapidly absorbed by the intestinal tract and activate PPARγ in the intestine, thereby alleviating sepsis‐induced acute intestinal injury (Tang et al. 2023a, 2023b). In this study, OA‐rich EN further activated PPARγ, inhibited STAT‐1 phosphorylation, promoted STAT‐6 phosphorylation, reduced iNOS expression, and up‐regulated the expression of Arg‐1, Fizz 1, and Ym 1 compared with EN alone. Through blocking the activation of PPARγ via the PPARγ‐specific antagonist SR202, the impact of OA‐rich EN on remodeling the M1/M2 polarization balance and alleviating the symptoms of IBD was reversed. Similarly, the cell study confirmed that SR202 could block the activation of PPARγ by OA and thus reverse its regulatory effects on the STAT‐1/STAT‐6 pathway and the M1/M2 polarization of macrophages. These results confirm that the protective effect of OA‐rich EN on IBD is dependent on the regulation of M1/M2 macrophage polarization via the PPARγ/STAT‐1/STAT‐6 pathway.

Several limitations exist in the present study. First, we only used DSS to induce an acute IBD model and did not investigate the effect of OA‐rich EN in a chronic model, which better reflects the pathological characteristics of clinical IBD. Second, in the experiments, the dose of OA‐rich EN was mainly based on the dose used in our previous studies on septic intestinal injury. The best therapeutic window and the safety of long‐term intervention remain to be further optimized. Third, the effects of OA‐rich EN on the intestinal flora have not been systematically studied. Intestinal flora dysbiosis is a key pathological feature of IBD, and bacterial metabolites (such as short‐chain fatty acids) are known to regulate intestinal immunity, maintain barrier integrity, and alleviate inflammatory responses, which may exert synergistic effects with OA.

## 5 Conclusion

This study is the first to confirm that OA‐rich EN could regulate the M1/M2 polarization of intestinal macrophages and simultaneously repair the intestinal mucosal barrier by activating the PPARγ/STAT‐1/STAT‐6 pathway, thereby significantly alleviating IBD‐induced intestinal injury. OA‐rich EN has the triple functions of nutritional support, barrier protection and immune regulation and may provide a new strategy for the clinical nutritional treatment of IBD. Further translational research will promote its clinical application in IBD patients, providing a new treatment option to enhance patient quality of life and lower disease recurrence rates.

## Conflicts of Interest

The authors declare no conflicts of interest.

## Data Availability

The data that support the findings of this study are available on request from the corresponding author.
